# The complexity of the dialogue between psychoanalysis, neurosciences, and genetics

**DOI:** 10.3389/fpsyt.2023.1084642

**Published:** 2023-01-20

**Authors:** Yorgos Dimitriadis

**Affiliations:** Université Paris-Cité, Centre de Recherches Psychanalyse, Médecine et Société, Paris, France

**Keywords:** psychoanalysis, neurosciences, psychiatry, genetics, complexity

## Introduction

The question of psychogenesis and organogenesis is among the oldest and—according to Ey (1)—the most complex chapters in psychiatry. At the start of the 19th century this debate was expressed through the opposition between functional and organic disorders. Other terms such as endogenous—most often opposed to exogenous or, alternatively, to reactionary, psychogenic, neurotic or even sociogenic—are almost simultaneous variants of the previous tandem (2, 3). In recent years, with the development of genomics and neuroscience, the etiology debate in psychiatry has tended to shift toward the interaction between the genome and environmental factors (the so-called “exposome”), as early on as the gestation period [see (4)]. A much more recent functionalist conception of the body-mind problem sees the brain as the hardware and the mind as the software, but Kendler (5) considers this dualistic conception to be an unfortunate combination of 19th century Cartesian neuropathology and computer functionalism. Nevertheless, the discoveries on neuronal plasticity [see (6)], with the observation that lived experience inscribes traces in the nervous system that can be re-combined with later experiences, provide the possibility to go beyond the opposition between psychic causality and organic causality. Thus, genetic and environmental factors could contribute in their own way to the configuration of these traces but also to their re-combination. Moreover, Crow has proposed (7) that “schizophrenia is the price Homo Sapiens pays for language”. Specifically, Crow has hypothesized that, for this very reason, the genes for schizophrenia must be related to the genes that make language possible. Moreover, in his 1946 Bonneval speech, Lacan argued that “Not only can man's being not be understood without madness, but it would not be man's being if it did not bear madness within itself as the limit of his freedom” [(8), p. 144]. Thus, views on psychopathology from fields as removed as genetics and psychoanalysis may unexpectedly echo one another.

## The complexity of genome-environment interaction in psychiatry

Kendler and Eaves (9) distinguished three main models (and their possible association) for the interaction between genotype and environment: additive effects of genotype and environment, genetic control of sensitivity to the environment, and genetic control of exposure to the environment. Thus, it would appear that genetic factors may sensitize or predispose individuals to exposure to particular environmental factors or even to experiences with depressogenic potential. For the same authors (9), genetics influences on human personality are probably related to traits such as impulsiveness, personal stability, and frustration tolerance. These traits could predispose to life experiences such as work and relationship changes which, in turn, can induce depression. Furthermore, genetic heritability in psychiatry is governed by complex mechanisms, since the same genes or the same genomic segments in copy number variation (CNVs) do not produce the same phenotypes and phenocopies can occur (these are environmentally-induced phenotypes which are identical to phenotypes that are genotypically determined in other individuals). Moreover, in psychiatry, heritability is not considered to be purely genetic and discoveries made since the 1980s through. Kendler suggests (10) that family twin and adoption studies (FTA) indicate that there is both environmental and genetic inheritance. Thus, “[t]he claim that FTA studies ‘prove' that a disorder is'biological' is weak. An astonishing wide array of human behaviors and traits, such as hours spent watching television, sports participation, church attendance […] are heritable” [(10), p. 1058]. He also claims (10) that a substantial proportion of genetic variation results from large numbers of small effect variants, each of which has a limited impact. Genome-wide association studies of schizophrenia (11–13) and manic-depression (13, 14) confirm this claim. Furthermore, with regard to autism, Munnich [(15), p. 89] argues that: “Since 2014, our exploration has included targeted sequencing of 250 genes known and published to cause autistic syndrome […] This approach has shown us the high frequency of accidental, genetic but non hereditary events […] there is no single autism gene, but dozens, even hundreds of distinct mechanisms that contribute to autism. Making the diagnosis of a genetic disease that presents as autism in no way precludes the use of psychotherapeutic approaches”. Even where genetic inheritance seems plausible, it is mostly non-specific and, furthermore, we cannot know whether what is inherited relates to specific nosological entities [which are, as Kendler (10) puts it, clinico-historical constructs] or a pathophysiological pathway. Which may predispose either to vulnerability to one or more clinical types or to one or more possible clinical expressions of the same. Moreover, Post and Weis (16) suggest that a particular genotype may predispose also to protective or adaptive mechanisms and other authors argue (as will also be seen below) that genes can predispose to creativity, which can be passed on alongside genes for vulnerability to certain psychopathologies.

## From a single cause to a multitude of possible causes

Kendler (17) claim that in the second half of the 20th century, psychiatry sought to distance itself from psychoanalytic domination and to re-establish its medical legitimacy through research into theories positing a single cause, much like Bayle [(18), see also (19)] had succeeded in isolating tertiary syphilis as the sole cause of general paralysis insane. For one thing, this complexity moves us away from the idea of a single causality and, for another, the plurality of causes means that each individual case is unique. As Ansermet and Giacobino [(4), p. 10] put it: “Rather than presiding over the repetition of sameness, genetic determinism poses the question of the production of difference. Inter-individual variations and the defining of singularity also become crucial questions for genetics”. What is more, “psychoanalysis involves the subject and its evermore unpredictable genesis, which relates to a gap between cause and effect that cannot be bridged” [(4), p. 74]. In this dialogue between genetics and clinical human sciences, the kind of psychopathology that takes into account the singularity of each case—namely, psychoanalytically-oriented psychopathology—surely has its place. In the light of neuroscience advances in neuroplasticity, the neuropathology of so-called mental illnesses should not abolish psychopathology. On the contrary, as is the case with psychosomatic diseases where pathological modification of tissues is a prerequisite for them to be qualified as psychosomatic, neuropathology does not contradict the “psychosomaticity” of so-called “mental illnesses”. Indeed, for Lacan (19), psychosomatic phenomena have to do with the gelification of the signifying chain: signifiers, being sometimes gelified, lose their signifying function and, as a result, become signals from the other and obtain an imperative value for the organism. Peirce's phaneroscopy helps us [see (20–23)] to conceptualize this as a process of semiotic reduction that moves from signifying tierceity to the secondness of the signal (which could condition or provoke reactions) or, even beyond, to the primacy of complete automatism. Even beyond the framework of classical psychosomatic phenomena, this process of semiotic reduction would be conceivable also for other clinical states related to homeostatic circuits of the brain, as I argued elsewhere regarding schizophrenia (20, 21), manic-depressive psychosis (22–24), depression (24), catatonia (25), addictions (26), traumatic neurosis (27), and panic disorder (20, 27).

The Freudian approach has always taken into account organic—genetic and epigenetic—factors in the determinism of psychological disorders. Freud certainly did not reject organicity as a cause. In fact in his concept of the *complementary series* (28), two factors converge in fulfilling an etiological requirement, and in his concept of *somatic complacency* (29), every hysterical symptom involves the participation of both sides, psychical and somatic, the latter offered by some normal or pathological process “in” or “connected” with one of the bodily organs. Hence, these two concepts were meant as a conversation with the psychiatric theories of his time—namely, the theory of the “constitution”. Lacan, for his part, far from excluding organic causes in the determination of psychoses and neuroses, referred to what could be a sensitization [(8), p. 182–83] or conditioning [(19), p. 207] of human soma.

Let us recall in this regard the sentence that closes Lacan's 1946 text [(8), p.182–83] on psychic causality: “I do not hesitate to say that it will be possible to demonstrate that the Oedipus crisis has physiological resonances—and that, however purely psychological it may be in its scope, a certain dose of Oedipus may be considered as having a humoral efficacy of the absorption of a desensitizing drug”. And the action of psychotropic drugs—which we could describe, at least in several cases, as desensitizing processes [see (22, 30)]—can resonate with the much more complex and most efficient desensitizing processes that psychoanalysis allows us to observe. Indeed, an infant's soma is conditioned by the signals of her primordial Other, that is until she becomes desensitized to these signals, through the triadic—mainly linguistic—processes inherent in ternary semiotics, the properly human semiotics. Nevertheless, sometimes, as a described above, through a “semiotic redaction”, produced both by psychic and biological predispositions, the human body may once again become attuned to signals that induce “automatic states”. Affects for instance—which rely on the metaphorical and metonymic processes of the unconscious—may be reduced to automatic emotions and/or moods (20–24, 27)—which loop and sustain themselves. Besides, in a letter to Fliess dated 1 January 1896 (31) Freud (K manuscript) recognizes heredity as a further determining factor, in that it promotes and increases pathological affect. Furthermore, Freud (32) in his paper, of the same year, “Heredity and the Aetiology of the Neuroses” gave also an important role in heredity regarding the causality of psychic suffering.

## An opportunity of renewed dialogue between psychoanalysis and psychiatry

The dialogue between psychiatry and psychoanalysis is by no means incompatible with neuroscientific and genetic studies. On the contrary, such interaction may reveal points of inertia in subjectification. Indeed, organic factors—amongst others—may interfere with the abovementioned desensitization and, consequently, with the subjectification of a given individual insofar as they interfere with his or her interactions with others, especially in the early years, which we know from psychoanalysis that they are foundational years. This is why intensive psychoanalytically inspired screening and psychotherapeutic work during these early years is crucial [e.g., (33, 34)]. Developmental psychology and neurodevelopmental studies also engage on this subject, with the concept of critical (or sensitive) periods [e.g., (35)] which allows precisely to bring new lights to the interaction genes/environment. Recent studies [for an overview see (36)] claim that there are neurobiological mechanisms (genetically determined) which regulate the impact of the environment during some precise “time windows” and also that these mechanisms can themselves be modified by the impact of certain environmental stimuli [for an discussion on this issue including psychoanalytic theory with a focus on schizophrenia see (36)]. However, the so-called “desabilitys” that can emerge when there are barriers to the knotting of the body with the symbolic, should not be reduced to deficiencies as they appear in the latest psychiatric classifications as psychiatric syndromes of the “neurodevelopmental disorders” category. The scientistic spirit of these taxonomies disregards the subjectivity of the so-called “mentally ill”. Indeed, their “desability” is primarily an interpersonal issue which must be addressed by clinicians who understand the meaning of “madness”. This should only additionally involve rehabilitative and medicinal treatments.

Biological psychiatry can interact with psychoanalysis either synergistically or antagonistically. In many cases of psychosis and autism neuroleptics can reduce the alienation felt by patients and, thus, facilitate transference and psychotherapeutic work and rehabilitation. This can typically happen in cases of coenesthetic and/or sensory invasion, pervasive feelings of persecution or states of agitation that, in the words of Czermak (37) (for person suffering from psychosis), render transference “irresistible” and “traumatic”. On the other hand, the immoderate use of antidepressants (38), neuroleptics (39), and lithium (40, 41) can, in a number of cases, result in losing motivation to address important issues, or even in emotional apathy, anhedonia, decreased libido and attenuation of creative capacities.

## On the genetic predisposition that is common to certain psychopathologies and creativity

Jamain [(42), p. 311] claims that epidemiological studies (43) regarding “Bipolar disorders” allow us to estimate that the proportion of “the disease explained by genes is about 60–80%, whereas the effect of the environment would contribute to about 40% of the disease” [see also (11, 14)]. According to Masson and Brun (44), the basis for “manic-depressive illness” is the pathological drop in the threshold of emotional reactivity, making also reference to Ey (45) who wrote about in his *Psychiatric Studies* as early as the 1950s. According to Masson and Brun (44), the emotional hyperreactivity that results from cerebral dysfunctions can be amplified by certain traumatic, behavioral, or toxic phenomena that trigger critical episodes. However, several studies have documented the possibility of a parallel genetic transmission of the propensity for creativity or certain forms of intelligence and vulnerability to certain clinical types. For example, manic-depressive psychosis is reported to be much more common in writers, especially poets (46–49).^1^ According to Andreasen (46) creative individuals have a particular cognitive style that both predisposes them to creation and leaves them vulnerable to thymic fluctuations. In this study, the predisposition to creativity also concerned their “healthy” relatives whose creativity was not otherwise limited to writing. Of course, the mechanisms of the correlation between madness and creativity are far from being elucidated. Is there a common mechanism for both? Or is it rather an effect of unusual experiences—as might also happen under the influence of psychedelic drugs—or even an attempt to synthesize divergent states or stimuli^2^ that push toward creation. Probably there is more than one cause here, but as we shall see in the conclusions, psychoanalytic theory has something to say about this issue.

## Discussion

The fact that all of us harbor something that is “out of the norm”, a form of “madness”, is by no means something to take offense at, but rather it allows us to relate to what is abnormal and specific about each individual—both the suffering or even deficits, and the originality and inventiveness. It has been established that the same genetic variants can predispose to symptoms but also to original creativity, and even, as Baron-Cohen et al. (53) suggest in the case of autism, to talents in fields that require systematization.^3^ As Malafosse also concludes, “it is possible that certain susceptibility alleles of psychiatric disorders constitute an advantage for the people who carry them and for society as a whole” [(54), p. 48]. Creativity and psychopathology are in no way incompatible, and one could even argue that many times is quite the opposite….

Since Freud, there have been discoveries about artistic creations by means of sublimation (55) and about delirium as a healing process (56). More recently, Lacan contributed his concepts of “suppléance” (supplementation) (57) and the “sinthome” (55). According to what Lacan says in 1975 (58) about James Joyce's writing style, language [which meant to have the function “not to inform but to evoke” [(59), p. 247], as he claimed back in 1956] has the main property to make us enjoy, beyond any sense—even equivocal—at the same point that it remains, according to Soler, “enigmatic” [(60), p. 18]. Scientists (61) have recently found that the language of apes is more “robust” than that of humans, but the latter is more efficient, “enabling better exploitation of information capacity and complex use of the neural vocabulary to adapt and learn new environments [(61), p. 606] […] In turn, it can contribute to fragility underlying human psychopathologies” [(61), p. 596]. In my opinion, human language is apt to “deviate” in order to “treat” the enigma of the social Other's desire. This again brings us back to the relationship of language to psychosis, at the genetic level. And to conclude, as Lacan [(62), p. 477] noted: “the only organicity that is essentially involved in this process: the organicity that motivates the structure of signification”.

## Author contributions

The author confirms being the sole contributor of this work and has approved it for publication.

## Footnotes

^1^For other authors such as Sclesinger (50) the association between creativity and “mad geniuses” is overstated in the professional and popular press.

^2^For which the terms “janusian process” (51), “overinclusive thinking”, and “cognitive inhibition” (52) have been used.

^3^This particular intelligence would be linked to sensory hypersensitivity and hyper-systematization.
